# Synapse development and maturation at the *drosophila* neuromuscular junction

**DOI:** 10.1186/s13064-020-00147-5

**Published:** 2020-08-02

**Authors:** Vivian T. Chou, Seth A. Johnson, David Van Vactor

**Affiliations:** Department of Cell Biology and Program in Neuroscience, Blavatnik Institute, Harvard Medical School, Boston, MA 02115 USA

**Keywords:** *Drosophila melanogaster*, Synapse, Neuromuscular junction, Bouton addition, Synaptic plasticity, Presynaptic active zone, Cell-adhesion molecules, Trans-synaptic signaling

## Abstract

Synapses are the sites of neuron-to-neuron communication and form the basis of the neural circuits that underlie all animal cognition and behavior. Chemical synapses are specialized asymmetric junctions between a presynaptic neuron and a postsynaptic target that form through a series of diverse cellular and subcellular events under the control of complex signaling networks. Once established, the synapse facilitates neurotransmission by mediating the organization and fusion of synaptic vesicles and must also retain the ability to undergo plastic changes. In recent years, synaptic genes have been implicated in a wide array of neurodevelopmental disorders; the individual and societal burdens imposed by these disorders, as well as the lack of effective therapies, motivates continued work on fundamental synapse biology. The properties and functions of the nervous system are remarkably conserved across animal phyla, and many insights into the synapses of the vertebrate central nervous system have been derived from studies of invertebrate models. A prominent model synapse is the *Drosophila melanogaster* larval neuromuscular junction*,* which bears striking similarities to the glutamatergic synapses of the vertebrate brain and spine; further advantages include the simplicity and experimental versatility of the fly, as well as its century-long history as a model organism. Here, we survey findings on the major events in synaptogenesis, including target specification, morphogenesis, and the assembly and maturation of synaptic specializations, with a emphasis on work conducted at the *Drosophila* neuromuscular junction.

## Background

The human brain contains approximately 100 billion neurons [1, 2] that are connected through trillions of synapses [3, 4]. Thus, understanding synaptogenesis—the process by which synaptic connections develop and mature, allowing neurons to form circuits that underlie perception, cognition and behavior—remains a monumental task. Chemical synapses are, with few exceptions, specialized asymmetric junctions that link a presynaptic neuron and a postsynaptic target [5, 6]. In humans, synaptogenesis begins primarily during embryonic development [7]. Synaptogenesis follows the process of axon pathfinding, whereby a motile structures known as growth cones at the axon tip navigate through a complex and dynamic environment to make physical contact with their proper targets by responding to a series of extracellular guidance cues [4, 8, 9]. Once axons arrive at their destinations, synapse formation is initiated through various adhesive interactions via cell-adhesion molecules (CAMs), substrate-adhesion molecules (SAMs), and bi-directional signaling between the pre- and postsynaptic compartments [5, 7]. As the synapse matures, the presynaptic axon assembles the machinery that mediates efficient neurotransmitter release via membrane fusion of synaptic vesicles (SV). SV release is triggered by an influx of Ca^2+^ ions via voltage-gated channels and occurs within specialized regions known as active zones (AZ) [7, 10–14]. Concurrently, the postsynaptic compartment, which may be the dendrite of another neuron or another cell type, accumulates neurotransmitter receptors within specialized regions that form in precise alignment with the presynaptic release sites [15–17].

**Fig. 1:**
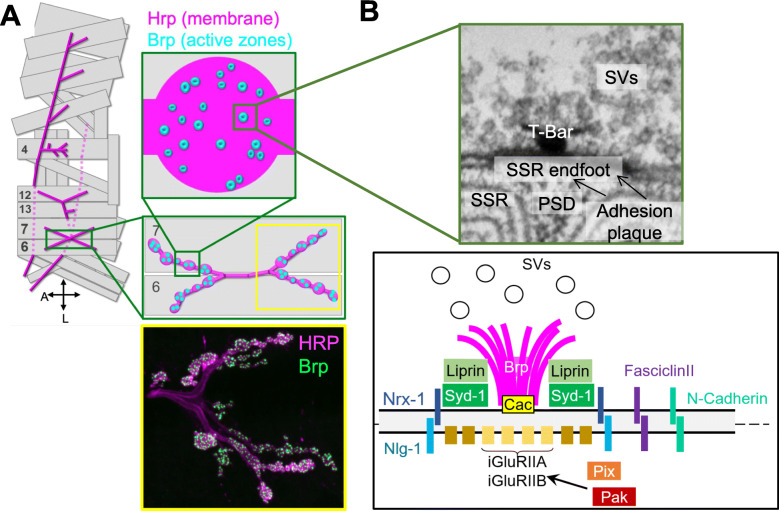
Organization of the pre- and postsynaptic cytomatrix. **a** Drawing of the larval musculature, cartoons of type I NMJ/boutons and immunostaining of NMJ branches in muscle 6/7 marked with α-Brp (green) for active zones and α-HRP (magenta) for overall shape. **b** (Top) Electron micrograph displaying presynaptic AZ components, including clustered SVs and the electron-dense Brp T-bar, as well as the membrane folds of the SSR, which is thought to be structurally and functionally analogous to mammalian dendritic spines. The expected localization of iGluRs is also indicated. (Bottom) Schematic of selected pre- and postsynaptic components. Communication between compartments is mediated by trans-synaptic interactions, such as the Dnrx1-Dnlg1 complex, through which the presynaptic AZ protein Syd-1 acts to regulate the maturation of iGluR receptors. GluR clustering is also mediated by dPak via Pix

Although understanding the glutamatergic synapses of the vertebrate central nervous system (CNS) is arguably one of the premier goals of neuroscience, PNS synapses of worms and flies, such as at the neuromuscular junction (NMJ), are particularly suitable models because they are tractable but also bear striking structural and chemical similarity to the synapses of the vertebrate CNS. Profound insights on the excitatory glutamatergic synapse in particular have emerged from studies of the *Drosophila* larval NMJ (Fig. 1) [18–22]. In the fly, the NMJ is a stereotyped structure that is easily accessible through dissection but also via non-invasive imaging through the translucent larval cuticle. More broadly, the *Drosophila* NMJ can be manipulated with sophisticated genetic mutagenesis, screening, expression, and editing strategies and is amenable to advanced biochemical, electrophysiological, ultrastructural, and light imaging techniques. Throughout synaptogenesis in *Drosophila*, conserved signaling pathways such as Bone Morphogenetic Protein (BMP) [23, 24], Wnt/Wingless (Wg) [25–27], Fibroblast Growth Factor (FGF) [28], and leukocyte common antigen–related receptor protein tyrosine phosphatase (LAR-RPTP) [29–32] coordinate the complex interplay of cellular and molecular events.

Synaptogenesis is not a singular event but rather a dynamic process. While the most intense period of synaptogenesis occurs during embryonic and early postnatal stages, nervous system development persists throughout adolescence and even into the third decade of human life [33]. Such changes encompass not only the addition of new synapses but also their “pruning” or removal; it is estimated that half of the synapses within the prefrontal cortex of human newborns are removed by adulthood [33]. Importantly, structural plasticity forms the basis of learning and memory and reflects the ability of synapses to respond not only to baseline developmental cues but to acute external stimuli [6, 34, 35]. This remarkable property of neurons is predicated on reciprocal bidirectional signaling and precisely orchestrated assembly and function of both the pre- and postsynaptic compartments [6, 34, 35], emphasizing the importance of understanding the synapse as an integrated whole.

## Synaptic specification: finding the right target

Upon physical contact of the axonal growth cone with its target, the specification and alignment of the correct pre- and postsynaptic partners is coordinated by various CAMs [7, 36–38]. CAMs act throughout synapse development, and beyond their roles in physical adhesion, also function as trans-synaptic signaling molecules and are involved in processes ranging from SV organization, receptor clustering, and structural and functional plasticity [36–38]. Key conserved regulators of CAM interactions include the trans-synaptic binding Eph receptor tyrosine kinases and their ligands, ephrins, which can both be expressed in either the pre- or postsynaptic compartment and facilitate bidirectional communication to regulate synaptic morphogenesis and plasticity [39, 40]. CAMs are divided into two classes: those that engage in homophilic interactions with another molecule of the same type, and those that engage in heterophilic interactions with a different CAM or to substrate adhesion molecules (SAMs) in the extracellular matrix (ECM) [36, 41]. CAMs are not necessarily limited to a single mode of binding. For instance, SynCAM [42–44] and the type II classical cadherins [45] display both homophilic and heterophilic binding in vivo, while type I classical cadherins show both forms of binding in vitro [46, 47], although only homophilic interactions are observed in vivo [48].

Conventionally homophilic CAMs include NCAM/CD56 [49, 50] and the related *Drosophila* protein Fasciclin II (FasII) [51–53]; DSCAM [54, 55]; SynCAM [42–44]; and the type I and II classical cadherins [56–59]. Of the homophilic CAMs, the type I cadherin Cadherin-N/Cadherin 2 (CadN) is the most highly expressed as well as best studied in the context of the CNS. CadN localizes within the synaptic cleft [60] and adjacent to presynaptic AZs [61] and redistributes in response to activity [62]. Beyond providing adhesive connections, CadNs are involved in neurotransmission [63], presynaptic short-term plasticity [64], dendritic spine morphogenesis [65, 66], activity-dependent plasticity and stabilization of dendrites [67, 68], and long-term potentiation [69, 70]. Other homophilic CAMs have similarly diverse functions, including neuronal morphogenesis [71–74], homeostatic [75] and activity-dependent plasticity [50, 53], organization of synaptic architecture [42, 76], and long-term potentiation [49, 77].

Heterophilic interactions at the synapse are mediated by CAMs such as Neurexins and Neuroligins [78–81], Teneurins [82, 83], and *Drosophila* Dprs and DIPs [84, 85]. The highly conserved Neurexins and Neuroligins are perhaps the best-characterized synaptic CAMs of all. Neurexins include three vertebrate members and a single fly homolog, Neurexin-1 (Nrx-1), while vertebrate Neuroligins 1–4 each have a corresponding *Drosophila* homolog (Nlg1–4) [78, 80]; additional homologs of both Neurexin and Neuroligin are present across various taxa. Neurexins and Neuroligins are both exclusively synaptic, and expression of either is sufficient to induce synaptic differentiation in vitro [86, 87]. Loss of either Neurexins or Neuroligins results in severe, or even lethal, defects in synapse formation and function in multiple systems [88–92], reflecting their central role in promoting the formation of stable junctions between pre- and postsynaptic components across organisms [88, 93–98]. Work at the *Drosophila* NMJ indicates that the postsynaptic targets of Nrx-1-Nlg1 include neurotransmitter receptors [93] and the WAVE regulatory complex, which modulates actin cytoskeletal dynamics [99]; downstream presynaptic targets of Nrx-1-Nlg1 include core AZ components [93] and BMP pathway receptors and effectors [94]. Further evidence for the bidirectional nature of Nrx-1-Nlg1 complex-mediated interactions include the finding that the presynaptic AZ component Syd-1 binds Nrx-1 directly to act via Nrx-1-Nlg1 to regulate GluRs composition and clustering [93]; this illustrates one mechanism by which the core AZ machinery can organize their postsynaptic counterparts. Moreover, loss of muscle Nlg1 results in defects in both presynaptic morphology and AZ assembly, indicating a reciprocal mechanism by which the postsynaptic apparatus can regulate release sites [93]. Interestingly, Nlg1 also regulates BMP pathway receptor subunits Wit/Tkv and the Mad [94], in a retrograde manner, further underscoring the bidirectional nature of Nrx-1-Nlg1 function.

It is worth noting that *Drosophila* motor neurons also express a protein known as Neurexin IV (Nrx-IV) [100] that is more properly classified as an ortholog of the contactin-associated protein (CASPR)/paranodin/CNTNAP family of receptors implicated in human autism-spectrum disorders [101, 102]. Although structurally similar from the C-terminus through the transmemembrane peptide and membrane proximal LamG repeats, this CASPR/CNTNAP branch of the Nrx family tree has one fewer LamG domains, and two additional extracellular domains (the FIB motif and N-terminal DISC domain). Nrx-IV is best known in fly for its vital role in septate junctions formed between glial cells to create an impermeable barrier between the CNS and the hemolymph [103]. However, Nrx-IV also participates in glial-neuronal interactions in multiple contexts [104–106]. Interestingly for this review, Nrx-IV plays an unexpected role *Drosophila* synapse biology, as alterations in presynaptic Nrx-IV expression levels results in abnormal NMJ morphology and AZ density ﻿synaptic morphology [107]. These functions of Nrx-IV are thought to be downstream of the micro (mi) RNA miR-34, which coordinates both pre- and postsynaptic synaptogenesis [108]. So far, it is not known whether this role for Nrx-IV is mediated by postsynaptic Nlg, or previously identified Nrx-IV receptor/co-receptors.

In comparison to decades of work on CAMs such as CadN and NCAM/FasII or the Neurexins and Neuroligins, recognition of the importance of Tenurins and Dprs/DIPs has been relatively recent. The highly conserved Teneurins include four vertebrate members, one *C. elegans* member, and two *Drosophila* members [82]. Vertebrate Teneurins have been best characterized for their roles in ﻿partner matching and cellular specificity in the visual system [109]. At the fly NMJ, presynaptic Ten-a and postsynaptic Ten-m form a heterophilic complex that regulates synaptic specification, neuronal morphology, synaptic architecture, and neurotransmission [110]. Notably, loss of neuronal *ten-a* and muscle *ten-m* produces defects in not only their respective compartment but also in the apposing cell: *ten-a* phenotypes include reduction in postsynaptic spectrin and the subsynaptic reticulum (SSR), while *ten-m* phenotypes include disruptions in presynaptic MTs, SVs, and T-bar morphology [110]. These findings demonstrate that Teneurins, along with Neurexin and Neuroligins, can mediate communication across the synaptic cleft in both anterograde and retrograde directions.

The trans-synaptic binding and downstream output of CAMs, in concert with other signaling cues and interactions with the ECM, orchestrates membrane contacts between pre- and postsynaptic partners. Expansion of the fly NMJ occurs through the Wnt/Wg- [111] and BMP-dependent [112, 113] addition of new boutons. Possible modes of bouton addition include asymmetric budding, similar to yeast cell division; symmetric bouton division; or de novo formation from the axon shaft [114]. Besides bouton addition, bouton elimination or pruning is also critical to refinement of synaptic structure and preventing overexuberant growth [18, 19]. Intermediary structures such as presynaptic “debris” and filopodia-like synaptopods are also observed during synaptic expansion, although unlike boutons, such structures are only observed very transiently [18, 19, 111, 115]. These sequential morphological processes of bouton addition, followed by expansion to full size and if necessary, pruning, are modulated by baseline and/or activity-dependent signaling cues to achieve a synaptic size and structural that facilitates proper connectivity and strength.

## Rapid initiation of presynaptic AZ assembly

During the morphogenesis of the larval type I bouton (both Ib and Is; refer to [116, 117] for detailed discussion of synapse and bouton types), initial outgrowth of membrane precedes recruitment of functional and architectural synaptic components. In support of this, numerous light imaging experiments in *Drosophila* have revealed that nascent boutons initially lack pre- and postsynaptic specializations [111, 118, 119]. Subsequent correlated timelapse light and ultrastructural studies in flies have shown that these immature “ghost” boutons are highly transient, as AZ precursors and SV docking become visible within minutes. Postsynaptic specializations are slower to form however with some components taking hours to arrive and fully accumulate [120, 121]. These processes of bouton addition and maturation can still be observed in cut axons, albeit at a lower frequency than in intact preparations, indicating local machinery is sufficient to support at least some level of synaptic expansion in the absence of protein synthesis [119].

The accumulation of AZ components produces a dense matrix of proteins, of which at least five are extensively conserved across multiple taxa: ﻿RIM/Unc-10, Munc13/Unc13, RIM-Binding Protein (RBP), Liprin-α/SYD-2, and ELKS/CAST/ERC/Bruchpilot (Brp) [11, 12]. Recent data suggests that while the AZ protein Syd-1 was formerly thought to be exclusive to invertebrates [122–124], new findings reveal a mouse homolog called MSYD1A [125]. However, when these components are viewed with ultrastructural resolution, the morphology varies across synapses and organisms (reviewed [11, 13]. For instance, *Drosophila* NMJs display a T-bar structure at the center of a larger an adhesion plaque characterized by electron-dense membrane morphology and a ladder-like set of repeated membrane-membrane cross-bridges (Fig. 1). In vertebrates, a grid-like arrangement of pyramidal dense projections have been described at the CNS [126], and sensory synapses display “ribbon” projections [127], which can range from flattened to spherical structures [128]. Notwithstanding these morphological variations, the accumulated evidence suggests that these dense projections have fundamentally similar roles in the function and organization of the presynapse; among these roles is the physical tethering of SVs via filaments to these projections, as has been observed in mice [129], frogs [130], and flies [131].

Despite the existence of local assembly mechanisms within the axonal compartment [119], in general, AZ development relies on trafficking of AZ components, as well as SV precursors (SVPs) and other materials, via long-range motor transport from the cell body [132–136], although mechanisms such as diffusion may also contribute [137, 138]. Several models exist for how AZ and SV components are organized into functional structures at release sites. Ex vivo studies have suggested the possibility that specialized dense core vesicles (DCVs) known as Piccolo/Bassoon Transport Vesicles (PTVs) traffic components in a unitary/quantal manner, such that each PTV contains a pre-assembled AZ “packet” [139, 140]. It has been proposed that AZ and SV materials are co-transported in aggregates of 1–2 PTVs and 5–6 SVPs that can very quickly form a functional AZ upon delivery [141–145]. However, the existence of PTVs and ready-to-go AZs packets has not been conclusively established through in vivo studies [146, 147], and to date, there is no evidence for PTVs containing pre-assembled AZs at the *Drosophila* NMJ or other invertebrate synapses, although interestingly, lysosome-related vesicles (PLVs) have been found to transport SVs and AZs in the *Drosophila* motor neuron axon [148].

## Early AZ assembly: SYD-1, LIPRIN-Α, and UNC-13

**Fig. 2:**
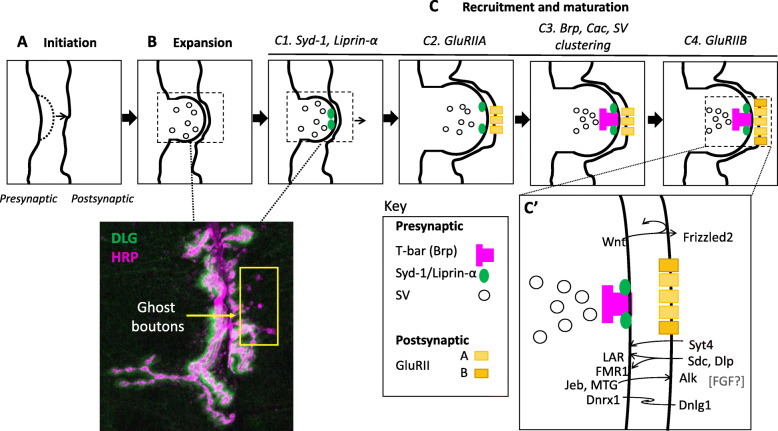
Overview of bouton growth and synaptic maturation. Addition of boutons is initiated by membrane outgrowth (**a**), followed by size expansion (**b**). (Top) Within minutes, AZ precursors are formed as early components such as Syd-1 and Liprin-α accumulate. (Bottom) Immunostaining of type I NMJ marked with α-Dlg for post synaptic SSR and α-HRP for presynaptic area. Immature boutons lacking postsynaptic structure (ghost boutons) are indicated (**Ci**). Maturation occurs as remaining components of the pre- and postsynaptic specializations are recruited, including GluRIIA-type receptors (**Cii**); Brp, Cac, and SVs (**Ciii**); and GluRIIB-type receptors (**Civ**). Inset (**c**′) shows bidirectional trans-synaptic pathways that are known to be important for synaptic development. Major pathways include both canonical and non-canonical Wnt and BMP signaling, while pathways mediated by Syt4, LAR, FMR1, Jeb, and MTG have also been described. The FGF pathway has also been reported, but pathways details, e.g. directionality, have not been defined

In contrast to the above model of pre-assembled AZs, in vivo studies in invertebrates suggest that while AZ assembly occurs very rapidly, a sequence of steps can still be distinguished (Fig. 2c) [146]. In support of hierarchical AZ assembly, ample studies in *C. elegans* and *Drosophila* indicate that two of the earliest components to be recruited to presynaptic AZs are the scaffolding proteins Syd-1/SYD-1 and Liprin-α/SYD-2 (*sy*napse-*d*efective) [93, 122–124, 149–154]; evidence in flies suggests that these components may precede other AZ proteins by hours [122, 149]. In both worms and flies, Syd-1 and Liprin-α have close spatial as well as functional relationships in driving early AZ assembly [122, 124] as well as SV organization [152, 154]. In flies, loss of *syd-1* and *liprin-α* results in reduced NMJ size as well as increased AZ size as observed by light and electron microscopy, respectively [120, 122], indicating potential defects in AZ organization. Furthermore, loss of either *syd-1* or *liprin-α* in *Drosophila* leads to decreased neurotransmission [120, 122].

The Syd-1 scaffold protein localizes to punctate clusters in presynaptic terminals and appears to be nervous-system specific in worms [123, 124, 155], as well as in flies [122]. Early studies established that Syd-1 has PDZ, C2 and RhoGTPase-activating protein (GAP)-like domains [155], although the RhoGAP activity of Syd-1 was long disputed and was only recently discovered to be required for the clustering of ELKS/Brp [153]. Syd-1 is potentially one of the earliest AZ components to be concentrated at the nascent AZ, as Syd-1 is upstream of Liprin-α in *C. elegans* [123, 124] as well as in *Drosophila*, where Syd-1 is required for proper Liprin-α accumulation [122]. Syd-1 also interacts with the Nrx-1-Nlg trans-synaptic adhesion complex via direct binding to Nrx-1 to regulate postsynaptic receptor clustering [93]. To date, no unambiguous mammalian homologs of Syd-1 have been identified, although mouse MSYD1A has been suggested as a possible homolog on the basis of partial sequence similarity and comparable roles in SV docking and synaptic transmission [125].

In contrast to Syd-1, the Liprin family member Liprin-α is highly conserved across metazoans, with ~ 50% similarity between the human and *C. elegans* homologs, and is widely expressed in many tissues in addition to the nervous system [121, 156]. Liprin-α has been well-characterized in the presynaptic compartment, and interestingly, has also been found to have a highly abundant postsynaptic expression, likely indicating postsynaptic roles [121, 156]. Structurally, Liprin-α contains an ﻿N-terminal coiled-coil region that mediates homo- and hetero-multimerization and an C-terminal Liprin homology (LH) region containing three SAM (sterile-α-motif) domains [120, 157, 158]. The SAM domains of the LH region, in particular, are thought to mediate the interactions of Liprin-α with Syd-1 and at least a dozen other proteins involved in synaptic development and/or function [121, 156]. Indeed, Liprin-α was first identified from its interactions with a member of the LAR-RPTPs, including vertebrate ﻿LAR, PTPδ, and PTPσ [157, 158]; while these initial observations were at the focal adhesions of non-neuronal cells, studies of LAR as well as Liprin-α have since focused on their diverse functions in the nervous system. LAR, in particular, has critical roles in axon guidance, neurite extension, as well as synapse assembly, formation, and plasticity [29, 30]. The Liprin family proteins also include Liprin-β, which, like Liprin-α, is required to promote synaptic expansion at the fly NMJ, and Liprin-γ/KazrinE, which antagonizes the function of Liprin-β at the NMJ [151]. However, the roles of Liprin-β and Liprin-γ remain largely unknown in comparison to Liprin-α, and it remains to be seen whether they have roles at the AZ.

A recent study in *Drosophila* has shown that Unc13A, one of the two fly isoforms of Unc13, a central regulator of neurotransmitter release, may be co-recruited with Syd-1 and Liprin-α [103]. Unc13 was first identified in *C. elegans* [104], with subsequent studies demonstrating its role in SV docking, priming, and fusion [159, 160]. Unc13 primes the SNARE/SM machinery for exocytosis [105, 161] and regulates the kinetics of release [103]. Through its regulation of SV release, Unc13 is also involved in diverse forms of plasticity, including short-term augmentation and long-term potentiation and depression [106, 162]. Super-resolution microscopy has revealed that the two *Drosophila* isoforms, Unc13A and Unc13B, occupy distinct localization patterns relative to other core AZ components and to Ca^2+^ channels, suggesting that different isoforms may act via independent pathways to tune and optimize SV release [103]. Interestingly, the shift from recruitment of Unc13A to Unc13B that occurs as AZ assembly and maturation progresses is reminiscent of a model where distinct receptor subunits in the postsynaptic compartment are recruited in a sequential manner (see the section “The Postsynaptic Cytomatrix” for more detail on this process; **Figure 2Cii-iv**) [93], perhaps reflecting separate roles of individual molecular isoforms at various stages of synapse maturation.

## Downstream AZ assembly: rim, rim-BP, and BRP

Following recruitment of Syd-1 and Liprin-α [122–124, 149], and the Unc13A isoform [103], the remaining AZ components are localized, including Unc13B, RIM/Unc-10, RIM-BP, and ELKS/Brp. Like Unc-13, RIM (*R*ab3-*i*nteracting *m*olecule) was first discovered as a regulator of SV release and neurotransmission [163, 164], and was later found to promote SV priming by monomerizing Unc-13 from autoinhibitory homodimeric complexes [165–167]. RIM also has roles in both short- and long-term plasticity [168, 169] and Ca^2+^ channel localization to the AZ [167, 170–172]. In particular, the regulation of Ca^2+^ channels by RIM is mediated by its interactions with RIM-BP [173, 174]. Together, RIM and the RIM-BP scaffold protein form a complex that interact with Ca^2+^ channels [172, 175]. The importance of RIM-BPs has been further demonstrated in *Drosophila,* where mutations in *rim-bp* results in defects in Ca^2+^ channel clustering as well as disruptions in Brp distribution, AZ ultrastructure, and synaptic release [176].

**Fig. 3:**
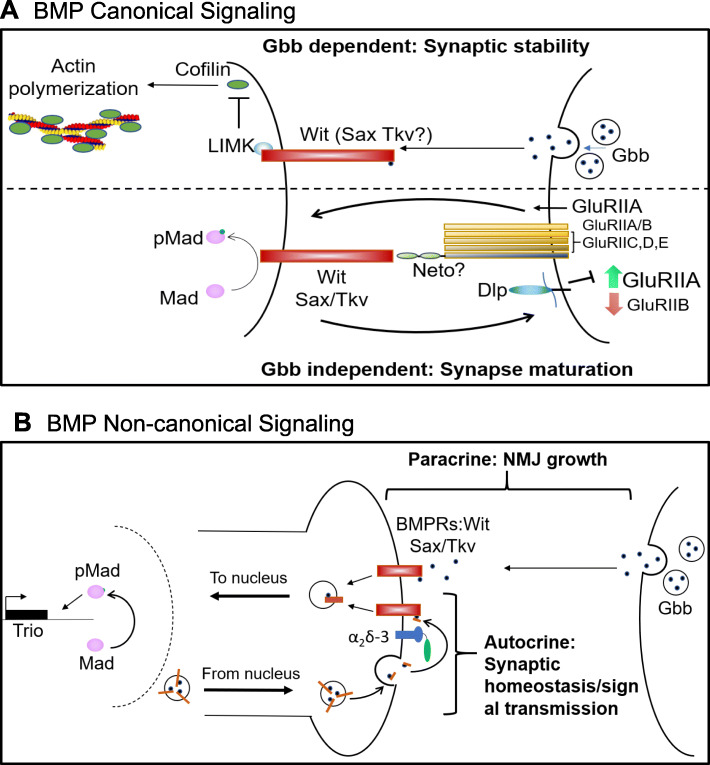
BMP Canonical and Non-Canonical signaling at the NMJ. **a** BMP canonical signaling includes a paracrine, retrograde mechanism whereby muscle-derived Gbb is released across the synapse where it activates BMPRs Wit and Sax/Tkv triggering internalization. Internalized Gbb is trafficked to the neuronal nucleus where it activates phosphorylation of the Mad to pMad to activate transcription of Trio-GEF promoting overall NMJ growth. A second autocrine canonical pathway involves release of Cmpy bound Gbb from the neuron where it sequestered by α_2_δ-3, a calcium channel subunit. Neuronally derived Gbb then binds BMPRs and causes transcriptional changes similar to the paracrine canonical pathway to regulate synaptic homeostasis and signal transmission. **b** BMP non-canonical signaling pathways include those that are Gbb dependent and independent. Gbb dependent non-canonical signaling involves retrograde release of Gbb where it is bound by Wit. LIMK acts as a Wit effector to inhibit Cofilin which normally promotes actin dynamics through filament severing. In a Gbb independent non-canonical mechanism, GluRIIA containing iGluRs form a positive feedback loop with local presynaptic pMad. This mechanism depends on Wit and Sax/Tkv, but not on transcription and is inhibited by Dlp (Glypican)

In addition to Unc-13, RIM, and RIM-BP, accumulation of the scaffold ELKS/Brp also follows initiation of AZ assembly by Syd-1 and Liprin-α, in a process that can begin within minutes of new bouton formation [119] but that can take hours to reach completion [149]. Work in *Drosophila* has provided compelling clues to the functions of Brp at the AZ. *Drosophila* Brp is expressed in two isoforms, both of which are necessary at the synapse [177]. Immunofluorescence has revealed that Brp forms distinct puncta in the presynaptic terminal of the motor neuron [178, 179] as well as in other neurons, such as R8 photoreceptors [180]. In live fluorescent studies, a nonfunctional truncated Brp-short construct [149, 181] has been frequently used as a marker of the presynaptic AZ, in lieu of the full-length Brp which can aggregate upon overexpression [179]. Importantly, Brp is an essential structural component of the AZ and is thought to be the major component of the electron-dense T-bar visible by electron microscopy (Fig. 3) [149], with loss of Brp resulting in the complete elimination of T-bars [178, 179]. Super-resolution stimulated emission depletion (STED) microscopy has revealed that Brp puncta are in fact donut-shaped structures that represent the top half of a funnel-shaped Brp complex which is attached to the membrane of synaptic release sites [149, 178], and that the two *Drosophila* Brp isoforms alternate in a circular pattern [177]. Further work combining direct stochastic optical reconstruction microscopy (dSTORM) super-resolution with electrophysiology demonstrated that mature AZs consist of approximately 137 rod-like Brp proteins organized into about 15 heptameric structures, and that proper neurotransmission relies on a precise maintenance of the stoichiometry and organization of this multimeric structure [182]. In addition to its role as a key AZ structural component, Brp is thought to regulate synaptic release by controlling the size of the readily releasable pool (RRP) of SVs [177]. Brp is also necessary for short-term plasticity and Ca^2+^ channel clustering [178], consistent with observations that the *Drosophila* Ca^2+^ channel subunit Cacophony (Cac) is recruited to the AZ contemporaneously [149].

The discussion in this review of AZ composition, architecture, and function addresses only a modest fraction of the expansive body of work on this highly complex and vital structure. The cytomatrix of the presynaptic AZ encompasses many other proteins, including: the scaffolds Piccolo/Fife and Bassoon; the Ras superfamily GTPases, such as Rab3 and Rac1, and their respective GAPs and guanine nucleotide exchange factors (GEFs); membrane trafficking machinery such as the SNARE proteins, synapsin, and synaptotagmin; the tripartite Cask/Mint1/Veli complex; developmental signaling pathways; and the cytoskeletal network [11, 12, 183–185]. Furthermore, despite significant progress in the field as a whole, many questions about the AZ remain. Ongoing and future studies will address topics such as the precise ultrastructure/nanostructure of the AZ, how the AZ is involved in both short- and long-term plasticity, and how the AZ senses and regulates synapse stability and integrity [11, 12].

## The postsynaptic CYTOMATRIX

In contrast to the presynaptic terminal, which is always neuronal [7], the postsynaptic target cell is most commonly neuronal but can also be of another cell type, such as glia [186–190] or effector organs, as in the case of the *Drosophila* NMJ. Early ultrastructural studies of dendritic spines of mammalian excitatory synapses detected a 3-dimensional intracellular structure known as the postsynaptic density (PSD) directly underneath the spine membrane [191–193]. The dendritic spine PSD, and analogous cytomatrix structures in other postsynaptic cell types [13], is rich in diverse protein types, including neurotransmitter receptors as well as scaffolding, signaling, and cytoskeletal molecules [15–17]. Glutamate receptors (GluR), the most prevalent receptor type, are present at excitatory glutamatergic synapses that predominate in the vertebrate CNS as well as the invertebrate NMJ [194, 195]. GluRs include ionotropic GluRs (iGluRs), an abundant subtype in which the receptors themselves form an ion channel pore to mediate fast synaptic transmission on millisecond timescales; this is in contrast to metabotropic GluRs (mGluRs), which activate ion channels via secondary messengers and are moreover slower-acting and less frequently occurring [194, 195]. iGluRs can be further subdivided into AMPA-, NMDA-, and kainate-type receptors, with AMPA and NMDA receptors being most common [194]. At the *Drosophila* NMJ, only AMPA/kainate receptors have been identified on the basis of genomic sequencing [195, 196]. However, more recent work has suggested that the iGluRs at the fly NMJ may be distinct from the classical vertebrate iGluR subtypes, as they display divergent structural and neurotransmitter-binding properties [197].

At the *Drosophila* NMJ, iGluRs are heterometric tetramers composed of three invariant subunits, GluRIII/GluRIIC, GlURIID, and GluRIIE, as well as one of either GluRIIA or GluRIIB [198–203]. Recruitment of iGluRs to the postsynaptic cytomatrix requires the function of the essential conserved auxiliary subunit Neto [204–208] and is thought to occur after the arrival of the presynaptic AZ components Syd-1 and Liprin-α but prior to the recruitment of Brp and Cac (Fig. 2c) [149]. GluRIIA and GluRIIB differ in their single channel properties, and they are responsible for large and small glutamate-gated currents, respectively [203, 209]. At the fly NMJ, proper iGluR subunit composition and GluRIIA/GluRIIB balance is regulated by Neto, likely via the recruitment of Pak [206], the *Drosophila* homolog of the p21-activated kinase (PAK) [210, 211]. The ratio of iGluRs containing the GluRIIA versus GluRIIB subunits is furthermore regulated by the presynaptic AZ protein Syd-1 via interactions with the Nrx-1-Nlg1 complex [93] and by a non-canonical, Smad-dependent BMP pathway [212]. This ratio of GluRIIA versus GluRIIB subunits changes throughout the lifespan of a synapse, with younger synapses preferentially incorporating GluRIIA, followed by GluRIIB incorporation as the synapse matures [181]. GluRIIA recruitment is thought to be an essential driver of synapse formation and its incorporation into the GluR tetramer is nearly irreversible [181, 209, 213]; this latter property, along with the tendency for GluRIIA incorporation to precede GluRIIB, likely accounts for the concentric arrangement of GluRs, where GluRIIB-rich receptors form a ring around GluRIIA-rich core (Fig. 2c) [93]. The proper temporal sequence of subunit recruitment, as well as relative spatial arrangement of GluRIIA- and GluRIIB-associated receptors is thought to be necessary not just for the initiation synaptogenesis but also subsequent stabilization and maturation [93].

Aside from neurotransmitter receptors, the postsynaptic cytomatrix is highly abundant in additional factors that play further roles in synaptic maturation and stabilization. Besides its above-mentioned roles in iGluR composition, Pak also modulates levels of the postsynaptic scaffold Discs Large (Dlg), muscle ultrastructure, and the formation of the SSR, a postsynaptic system of tubular-lamellar membrane folds that envelops the presynaptic bouton [214–217] that has been described as the structural and functional equivalent of dendritic spines at the *Drosophila* NMJ [218]. Another notable regulator of SSR expansion is Syndapin (Synd), a member of the F-BAR family of membrane-sculpting proteins [219, 220] that appears to exclusively postsynaptic where it likely mediates SSR development by regulating the muscle actin cytoskeleton [221, 222]. Development of the SSR also requires additional actin-regulatory proteins concentrated in the postsynaptic cytomatrix, including hu-li tai shao (Hts)/Adducin, an actin-capping protein [223–225], and Enabled (Ena) [226], which promotes the assembly of linear F-actin; other key cytoskeletal regulators of SSR morphogenesis include both pre- and postsynaptic Spectrin [227, 228] and presynaptic Ankyrin [229, 230]. Finally, Dlg, a PDZ domain containing protein, vertebrate PSD-95/SAP90, belongs to the membrane-associated guanylyl kinases (MAGUKs) family of PDZ proteins [15, 16] and promotes the clustering of Shaker K^+^ ion channels [231] and of FasII [232]. Dlg is also necessary for clustering of GluRIIB-containing receptors [233], while vertebrate PSD-95 regulates the clustering of NMDA receptors [234]. While Dlg is most abundant in the postsynaptic SSR, it is also present at lower levels in the presynaptic AZ [235], similar to the distribution of Liprin-α [121, 156].

Some factors traditionally associated with chiefly pre or postsynaptic roles including Dlg and Liprin-α, also display opposite localization albeit at a reduced abundance, suggesting the need to reconsider these factors for in new contexts. Dlg presents a presynaptic pool similar to Liprin-α which has been shown to promote the AZ localization of Cac, with effects on synaptic release probability and short-term plasticity [236]. Liprin-α, which has been chiefly studied as a presynaptic AZ protein, displays an overwhelmingly postsynaptic distribution [121, 156]. Postsynaptic Liprin-α interacts with the PDZ-domain protein GRIP in the PSD and is also regulated by the Ca^2+^/calmodulin- dependent protein kinase II (CaMKII) [237]. Outside of these studies, the roles of postsynaptic Liprin-α, including at the *Drosophila* NMJ, is largely a mystery, and may prove fertile ground for future inquiry. It is also interesting to consider the possible postsynaptic expression of other conventionally presynaptic proteins, such as Syd-1, which appears to display some postsynaptic staining [122].

## Trans-synaptic communication between compartments

Despite distinctions between pre- and postsynaptic compartments, effective bidirectional communication is essential to the coordinated assembly, maturation, and function of the entire synapse (Fig. 2c**’**). Key orchestrators of trans-synaptic communication include signal transduction pathways, including the Wnt/Wg pathway as a primary driver of anterograde signaling [25–27], and the BMP pathway as a major contributor to retrograde signaling [24]. These signaling pathways control many different aspects of neurodevelopment including growth, signal transmission, synaptic maturity, AZ assembly and homeostasis. Intriguingly, these many functions are controlled by signaling pathways that share many similar growth and downstream factors yet are capable of distinguishing between these functions as required during development. This section will detail the different categories and subcategories of signaling and describe how the developing NMJ is capable of discriminating between them.

At the fly NMJ, Wg participates in both anterograde and autocrine signaling. Wg is secreted from the presynaptic terminal and binds Frizzled2 (Fz2) receptor embedded in the postsynaptic membrane. Activation of Fz2 triggers internalization and cleavage of the receptor allowing a C-terminal fragment (Fz2-C) to enter the postsynaptic nucleus where it contributes to changes in gene expression thereby positively regulating synaptic expansion and plasticity [27, 238]. Retrograde Wg signaling is negatively modulated by several extracellular factors including extracellular matrix metalloproteinase enzymes (MMPs) and the decarboxylesterase Notum, which cleave HSPG co-receptors and Wg, respectively to limit signaling [239]. Presynaptic Wg ligand also mediates a divergent canonical pathway that regulates the presynaptic cytoskeleton. Neuronally derived Wg inactivates glycogen synthase kinase 3 (GSK3)/Shaggy, which in turn regulates microtubule dynamics through interactions with the Map 1B homolog Futsch [240–242].

BMP signaling controls many aspects of neuronal development at the NMJ including growth, synaptic transmission and synaptic homeostasis. Many of these functions of BMP signaling do not happen concurrently, yet use many the same factors to elicit these responses. How then do cells disentangle each of these signaling functions to achieve proper development? Recent exciting findings complement past studies to show how we can begin to separate these functions in terms of differences in signaling. Drosophila utilize both canonical and non-canonical BMP signaling. In the canonical BMP pathways, the Glass Bottom Boat (Gbb) ligand, most homologous to BMP7, is secreted by the muscle and binds with tetrameric complexes containing the type II receptor Wishful Thinking (Wit) and the type I receptors Thickveins (Tkv) and Saxophone (Sax). This interaction mediates the phosphorylation of the transcription factor Smad/Mothers against decapentaplegic (Mad) [243–245], which affect transcription leading to activity-dependent growth or synaptic clustering [113]. Canonical BMP signaling may also regulate actin through its interactions with the Rho-GEF Trio; it is thought that the retrograde BMP pathway regulates transcription levels of presynaptic Trio to restrict synaptic growth [246].

While the previous canonical BMP description represents an anterograde, transsynaptic mechanism, a second canonical BMP pathway involves an autocrine signaling mechanism where an activity-dependent stimulus directs synaptic strengthening and maturation [247]. Autocrine signaling occurs when an activity dependent stimulus promotes release and reabsorption of Gbb by the motorneuron, triggering a locally sustained BMP response. Recently, a calcium channel subunit (α_2_δ-3) was found to physically interact with neuronally-derived Gbb and was required to limit the range at which Gbb could work to strengthen the synapse [248].

Both paracrine and autocrine BMP canonical signaling utilize Gbb binding to Wit to cause a signaling cascade allowing pMAD to regulate transcription. How then does the motor neuron distinguish between these two signals to regulate two separable functions (bouton growth and synaptic (AZ) density)? Temporal requirements for each type signal partially explain the separability as the muscle-derived growth signal is primarily needed early phases (L1), while the sustained neuronally derived signal is required at later stages to refine and strengthen the synapse [248]. Additionally, factors that interact with Gbb can also separate functions. Crimpy (Cmpy) physically interacts with Gbb to promote recruitment of Gbb to dense core vesicles (DCVs) where it can be released from neuronal terminals with the Cmpy ectodomain [249]. Loss of Cmpy causes excessive growth, likely because without Cmpy, the neuron misinterprets autocrine BMP signaling for muscle-derived BMP signaling, which normally controls overall growth.

Several non-canonical BMP pathways have also been identified and be divided based upon whether they utilize Gbb. Non-canonical Gbb-dependent signaling controls bouton growth and occurs when, Gbb is secreted by the muscle and interacts with neuronal Wit to activate LIM Kinase1 (LIMK1). LIMK1 regulates activity-dependent bouton formation by promoting actin polymerization [112, 250]. Non-canonical Gbb-independent signaling promotes stability of GluRIIA subunits in the glutamate receptor heterotetramer. Gbb-independent non-canonical signaling involves a positive feedback loop whereby GluRIIA promotes accumulation of a local presynaptic pool of pMad at the AZ. pMad in turn serves to cluster and stabilize additional GluRIIA subunits preferentially over GluRIIB [212]. Importantly, this instance of BMP signaling utilizes a separate pathway from the canonical BMP signaling as the accumulation of synaptic pMad does not lead to NMJ overgrowth seen when nuclear pMad acts as a transcriptional regulator. Furthermore, while this positive feedback mechanism does require Wit and Sax, it does not utilize LimK, distinguishing it from non-canonical Gbb-dependent BMP signaling. If this pathway does not communicate through a diffusible factor like Gbb, how then is transsynaptic communication achieved? As pointed out in Sulkowski et al., the synaptic cleft is ~200A and GluR tetramer and BMP receptors (BMPRs) extend ~135A and ~ 55A, respectively into the synaptic cleft space. The GluR auxiliary protein Neto increases the span of the GluR tetramer suggesting it could serve as the transsynaptic link, though careful testing of this model is needed.

Besides Wnt/Wg and BMP, numerous other signal transduction pathways of consequence have been described at the fly NMJ. Multiple studies have established roles of retrograde signaling by Synaptotagmin 4 (Syt4) [251, 252] and LAR (Leukocyte common antigen related) [31, 120]. While Syt1, the other synaptically-expressed *Drosophila* synaptotagmin isoform, is associated with presynaptic SVs [253], Syt4 is a strictly postsynaptic Ca^2+^ sensor that regulates presynaptic SV fusion and activity-dependent structural plasticity in response to postsynaptic Ca^2+^ influx [251, 252]. Synaptic signaling is also regulated by heparan sulfate proteoglycans (HSPGs) such as Dallylike (Dlp) and Syndecan (Sdc), which impact multiple pathways including Wnt/Wg and BMP [254, 255]. Dlp acts as a negative brake on the positive feedback loop between GluRIIA and presynaptic pMad that is itself inhibited by presence of Octopamine, the Drosophila analog of nonadrenaline [256]. Additionally, Dlp and Sdc regulate the RPTP LAR, with effects on presynaptic neuronal morphology and AZ assembly [31, 120]. An open question is whether the interaction of these HSPGs with LAR results from a direct physical binding, or whether it involves another mechanism, such as secretion of a factor. Moreover, the effect of HSPGs on both LAR and BMP signaling, along with the observation that the Rho-GEF Trio is downstream of both LAR [257, 258] and BMP [246], raises the possibility of cross-talk between the two pathways. Dlp and Sdc have more recently been found to also act via the intracellular Fragile X Mental Retardation 1 (FMR1) protein in a retrograde manner in order to interface with the Wnt and Jelly Belly-Anaplastic Lymphoma Kinase (Jeb-Alk) signaling pathways [259]. The Jeb-Alk pathway is itself an anterograde trans-synaptic signaling pathways that regulates presynaptic morphology and neurotransmission [260] under the control of Mind-The-Gap (MTG), a presynaptically secreted molecule that modulates the extracellular environment of the synaptic cleft [19, 261–263].

Interestingly, the two *Drosophila* FGF receptors, Heartless (Htl) and Breathless (Btl), have been reported to interact with the spinal muscular atrophy-associated (SMA) protein Survival Motor Neuron (SMN) to regulates NMJ morphogenesis, consistent with the roles of FGF at vertebrate synapses [28, 264, 265]. Muscle-expressed Htl is itself required for presynaptic morphology, although the precise trans-synaptic mechanisms by which this is achieved remain to be defined [28]; nonetheless, observation that both SMN [266] and Htl [28] co-localize with the postsynaptic SSR raises the possibility that SMN functions downstream of FGF signaling in muscle. This interaction is intriguing given that SMN also appears to function downstream of the presynaptic BMP effectors Mad and Dad [266], suggesting that synaptic SMN may respond to distinct inputs in presynaptic and postsynaptic compartments. Indeed, SMN is known to be required on both sides of the synapse to support normal NMJ development and growth [28, 266–268] a property of multiple molecules that shape NMJ structure and function [226, 235, 269–272].

Many of the molecules and pathways involved in the communication mechanisms described above have well-established roles in regulating both bouton morphogenesis and AZ assembly, raising the possibility that these two processes are coupled. In fact, the morphological and AZ phenotypes associated with numerous Wg, BMP, and LAR pathway components are consistent with an inverse relationship between the assembly of the AZ and the size of presynaptic terminals as determined by bouton number. For instance, within the LAR pathway, this correlation is true for the LAR receptor itself as well as for Liprin-α [120], the HSPG ligand Dlp [273], the kinase Abelson (Abl) [274], the Rho-GEF Trio [153, 275], and the F-actin assembly molecule Ena [269]. The negative correlation between AZ and NMJ size is also observed for Syd-1, and its downstream effectors Nrx-1 and Nlg1; while Syd1 and Nrx-1-Nlg are not confirmed LAR interactors, they potentially interface with the LAR pathway through the interaction of Syd-1 and Liprin-α [93, 122]. While causative relationships and underlying mechanisms cannot yet be concluded, the accumulated evidence suggests that the regulatory machinery of bouton growth and AZ assembly are not independent. The coordination of morphogenesis and AZ formation may ensure the proper allocation of a finite pool of biological material; this is consistent with the finding that stoichiometric and homeostatic remodeling of the AZ dynamically tunes synaptic strength [276]. Thus, a conceivable explanation for coordination of these processes is that restricting AZ size ensures that all boutons are populated with release sites even as membrane expansion continues.

## Conclusions

At present, the homologs of 75% of all human disease genes have been discovered in *Drosophila* at nearly 600 loci, further underscoring the relevance of flies to human biology [277, 278]. This suggests that *Drosophila* can continue to be an excellent model for studying molecules and processes with close analogs at the human synapse while using tools and methods that may not be as readily available or effective in higher organisms. It can be expected that key insights on synaptic biology and connectivity will continue to emerge from studies of the larval NMJ, as well as additional synaptic contexts, such as the adult fly visual system and the NMJs of fly embryos and earlier-stage larva. The potential of model systems such as *Drosophila*, combined with ever-accelerating innovations in experimental and analytical techniques, presages exciting steps forward in tackling the fundamental questions of modern neurobiology.

## Data Availability

Not applicable.

## Abbreviations

Abl: Abelson
Alk: Anaplastic Lymphoma Kinase
AMPA: α-amino-3-hydroxy-5-methyl-4-isoxazolepropionic acid
AZ: Active zone
BMP: Bone Morphogenetic Protein
Brp: Bruchpilot
Btl: Breathless
Cac: Cacophony
CAM: Cell-adhesion molecule
CaMKII: Ca2+/calmodulin-dependent protein kinase II
CASPR: Contactin-associated protein
CAST: Cytomatrix at the active zone-associated structural protein
CNS: Central nervous system
Dad: Daughters against decapentaplegic
DCV: Dense core vesicle
DIP: Dpr Interacting Protein
DLG: Discs Large Dlp Dallylike protein
Dpr: Defective Proboscis Extension Response
Nlg: Drosophila Neuroligin
Nrx-1: Drosophila Neurexin
DSCAM: Down Syndrome Cell-Adhesion Molecule
dSTORM: Direct stochastic optical reconstruction microscopy
ELKS: Protein rich in the amino acids E, L, K and S
Ena: Enabled
ECM: Extracellular matrix
ERC: ELKS/RAB6-interacting/CAST family member
FGF: Fibroblast Growth Factor
FMR1: Fragile X Mental Retardation 1 protein
GABA: Gamma aminobutyric acid
GAP: GTPase-activating protein
Gbb: Glass Bottom Boat
GEF: Guanine nucleotide exchange factor
GluR: Glutamate receptors
GSK3: Glycogen synthase kinase 3
HSPG: Heparan sulfate proteoglycan
Htl: Heartless
Hts: Hu-li tai shao
iGluR: Ionotropic GluR
Jeb: Jelly Belly
LAR: Leukocyte common antigen-related
LIMK1: LIM Kinase1
Liprin: LAR-interacting protein
LH: Liprin homology
Mad: Mothers against decapentaplegic
MAGUK: Membrane-associated guanylyl kinases
Mav: Maverick
mGluR: Metabotropic GluR
MTG: Mind-The-Gap
Munc/Unc: Mammalian uncoordinated/uncoordinated
NMDA: N-methyl-D-aspartate
NMJ: Neuromuscular junction
PAK/Pak: p21-activated kinase
PDZ: PSD protein, DLG, zonula occludens-1 protein
PNS: Peripheral nervous system
PSD: Postsynaptic density
PTV: Piccolo/Bassoon transport vesicles
RIM: Rab interacting molecule
RIM-BP: RIM-binding protein
RPTP: Receptor protein tyrosine phosphatase
RRP: Readily releasable pool
SAM: Sterile-α-motif
SAM: Substrate adhesion molecules
Sax: Saxophone
Sdc: Syndecan
SM: Sec1/Munc18-like
SMA: Spinal muscular atrophy-associated
SMN: Survival motor neuron
SNARE: Soluble N-ethylmaleimide-sensitive factor activating protein receptor
SSR: Subsynaptic reticulum
STED: Stimulated emission depletion
SV: Synaptic vesicle
SVP: SV precursor
Syd: Synapse-defective
Syt: Synaptotagmin
Ten: Teneurin
TGF-β: Transforming growth factor β
Tkv: Thickveins
Wit: Wishful Thinking
Wnt/Wg: Wingless Int-1/Wingless
